# 6-Amino-8-(2-bromo­phen­yl)-1,7,8,8a-tetrahydro-3*H*-isothio­chromene-5,7,7-tricarbonitrile dimethyl­formamide solvate

**DOI:** 10.1107/S1600536809039105

**Published:** 2009-10-03

**Authors:** Hai-Yan Zhang, Jian-Rong Wu, Xiang-Shan Wang

**Affiliations:** a Xuzhou Jinmao Chemical Limited Company, Xuzhou Jiangsu 221002, People’s Republic of China; b School of Chemistry and Chemical Engineering, Xuzhou Normal University, Xuzhou Jiangsu 221116, People’s Republic of China

## Abstract

In the title compound, C_18_H_13_BrN_4_S·C_3_H_7_NO, the thio­pyran ring and the adjacent six-numbered ring adopt distorted boat conformations. The mol­ecules, lying about inversion centers, form hydrogen-bonded dimers involving one of the H atoms on the amino group with the N atom of a cyano group of an adjacent mol­ecule, resulting in a 12-membered ring system [*R*_2_^2^(12) ring motif]. The other H atom of the amino group forms an inter­molecular hydrogen bond with the O atom of the dimethyl­formamide (DMF) mol­ecule. Another lone pair of electrons on the same carbonyl O atom of DMF mol­ecule forms a non-classical C—H⋯O inter­molecular hydrogen bond, resulting in a chain of mol­ecules.

## Related literature

For the biological activity of related compounds, see: Karsten & Krisztina (2007 ▶); Wang *et al.* (1998 ▶, 2006 ▶); Zhang *et al.* (2008 ▶). For a related structure, see: (Mereiter *et al.* 2000 ▶). For graph-set notation, see: Bernstein *et al.* (1994 ▶).
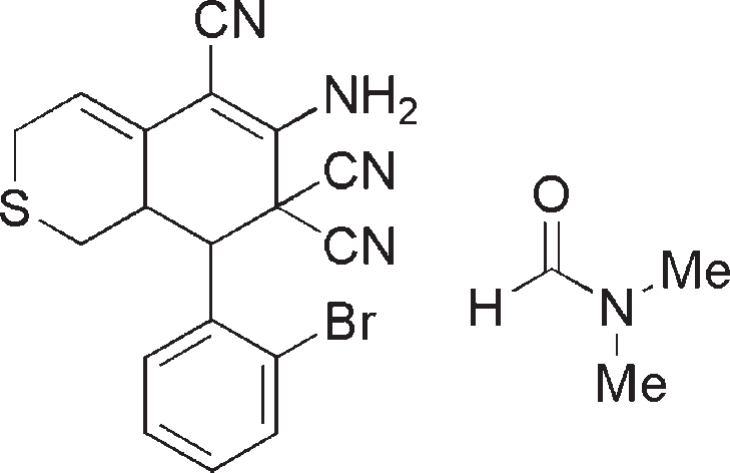


## Experimental

### 

#### Crystal data

C_18_H_13_BrN_4_S·C_3_H_7_NO
*M* *_r_* = 470.39
Monoclinic, 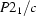

*a* = 14.7733 (4) Å
*b* = 9.1710 (3) Å
*c* = 15.7897 (4) Å
β = 92.478 (2)°
*V* = 2137.28 (11) Å^3^
*Z* = 4
Mo *K*α radiation
μ = 2.04 mm^−1^
*T* = 296 K
0.44 × 0.36 × 0.05 mm

#### Data collection

Bruker SMART CCD area-detector diffractometer
Absorption correction: multi-scan (*SADABS*; Sheldrick, 1996 ▶) *T*_min_ = 0.422, *T*_max_ = 0.900
13894 measured reflections
3841 independent reflections
2723 reflections with *I* > 2σ(*I*)
*R*_int_ = 0.032

#### Refinement

*R*[*F* ^2^ > 2σ(*F* ^2^)] = 0.036
*wR*(*F* ^2^) = 0.098
*S* = 1.04
3841 reflections
270 parameters
H atoms treated by a mixture of independent and constrained refinement
Δρ_max_ = 0.32 e Å^−3^
Δρ_min_ = −0.23 e Å^−3^

### 

Data collection: *SMART* (Bruker, 2001 ▶); cell refinement: *SAINT* (Bruker, 2001 ▶); data reduction: *SAINT*; program(s) used to solve structure: *SHELXS97* (Sheldrick, 2008 ▶); program(s) used to refine structure: *SHELXL97* (Sheldrick, 2008 ▶); molecular graphics: *SHELXTL* (Sheldrick, 2008 ▶); software used to prepare material for publication: *SHELXTL*.

## Supplementary Material

Crystal structure: contains datablocks global, I. DOI: 10.1107/S1600536809039105/pv2211sup1.cif

Structure factors: contains datablocks I. DOI: 10.1107/S1600536809039105/pv2211Isup2.hkl

Additional supplementary materials:  crystallographic information; 3D view; checkCIF report

## Figures and Tables

**Table 1:** Hydrogen-bond geometry (Å, °)

*D*—H⋯*A*	*D*—H	H⋯*A*	*D*⋯*A*	*D*—H⋯*A*
N3—H3*B*⋯O1^i^	0.89 (4)	1.96 (4)	2.856 (4)	178 (3)
N3—H3*A*⋯N4^ii^	0.79 (3)	2.54 (3)	3.294 (4)	162 (3)
C15—H15*A*⋯O1^iii^	0.93	2.54	3.438 (4)	162

Symmetry codes: (i) 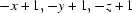; (ii) 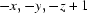; (iii) 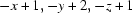.

**Fig. 1.**
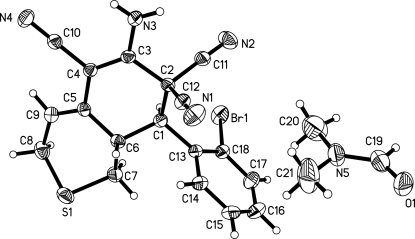
The molecular structure of (I) showing 50% probability of displacement ellipsoids and the atom-numbering scheme.

**Fig. 2.**
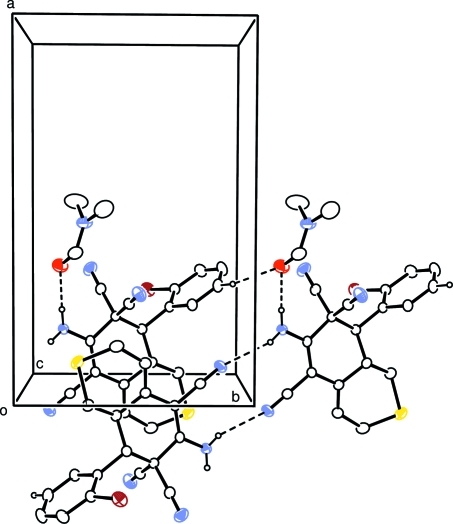
The molecular packing diagram showing the hydrogen bonds in the crystal of (I).

## supplementary crystallographic information

### Comment

Iso(thio)chromene derivatives are important and useful skeletons in organic synthesis. For example, it was reported that many isochromene derivatives displayed a wide range of biological activities, such as antiinflammatory activity (Wang *et al.* 2006), antitumor activity (Wang *et al.* 1998), antiviral activity (Karsten & Krisztina, 2007), and anti-apoptotic activity (Zhang, *et al.* 2008). We report here the crystal structure of the title compound, (I).

In the crystal structure of (I) (Fig. 1), the thiopyran ring adopts a distorted boat conformation: the atoms C5—C6, C8—C9 are coplanar, while the atoms C7 and S1 deviate from the plane by 0.332 (6) and -0.628 (6) Å, respectively. The adjacent six-numbered ring (C1—C6) also adopts a distorted boat conformation, with the atoms C1 and C6 deviating from the plane defined by atoms C2—C5 by 0.330 (5) and -0.404 (5) Å, respectively. The basal plane of the ring C1—C6 forms a dihedral angle of 16.5 (2) ° to the thiopyran ring and is nearly perpendicular to the benzene ring (C13—C18), forming a dihedral angle of 86.8 (1) °

The classical (N—H···O and N—H···N) and non-classical (C—H···O) inter-molecular hydrogen bonds are present in the crystal structure of (I). The molecules of (I) lying about inversion centers form hydrogen bonded dimers involving one of the hydrogen atoms (H3A) on the amino group with the atom N4 of the cyano group of an adjacent molecule, resulting in a twelve membered ring system which may be described in terms of graph set notation (Bernstein *et al.* 1994) as R_2_^2^(12) ring motif; details are given in Table 1 and Figure 2. The other hydrogen atom (H3B) of the amino group forms an intermolecular hydrogen bond with atom O1 of the DMF molecule. An other lone pair of electrons on the same carbonyl O1 atom of DMF molecule form a non-classical intermolecular (C15—H15A···O1) hydrogen bond, thus resulting in a chain of molecules.

The crystal structure of a closely related compound has been reported (Mereiter *et al.* 2000).

### Experimental

The title compound, (I), was prepared by the reaction of 2-bromobenzaldehyde (1 mmol, 0.185 g), malononitrile (1.2 mmol, 0.079 g) and 2-(tetrahydrothiopyran-4-ylidene)malononitrile (1 mmol, 0.164 g) in 1-butyl-3-methylimidazolium fluoroborate (20 ml) at 353 K. The single crystals suitable for X-ray diffraction were obtained by slow evaporation from a DMF solution.

### Refinement

The H atoms bonded to C atoms were included at geometrically calculated positions and in riding mode at C—H distances 0.93, 0.96, 0.97 and 0.98 Å for aryl, methyl, methine and methylene type H-atoms, respectively, with U_iso_(H) = 1.5U_eq_(methyl C-atoms) and 1.2U_eq_(non-methyl C-atoms). The H-atoms bonded to N3 were allowed to refine with isotropic displacement parameters.

### Crystal data

**Table:**

C_18_H_13_BrN_4_S·C_3_H_7_NO	*F*(000) = 960
*M**_r_* = 470.39	*D*_x_ = 1.462 Mg m^−^^3^
Monoclinic, *P*2_1_/*c*	Melting point = 530–532 K
Hall symbol: -P 2ybc	Mo *K*α radiation, λ = 0.71073 Å
*a* = 14.7733 (4) Å	Cell parameters from 4434 reflections
*b* = 9.1710 (3) Å	θ = 2.6–22.6°
*c* = 15.7897 (4) Å	µ = 2.04 mm^−^^1^
β = 92.478 (2)°	*T* = 296 K
*V* = 2137.28 (11) Å^3^	Plate, colourless
*Z* = 4	0.44 × 0.36 × 0.05 mm

### Data collection

**Table:**

Bruker SMART CCD area-detector diffractometer	3841 independent reflections
Radiation source: fine-focus sealed tube	2723 reflections with *I* > 2σ(*I*)
graphite	*R*_int_ = 0.032
φ and ω scans	θ_max_ = 25.2°, θ_min_ = 1.4°
Absorption correction: multi-scan (*SADABS*; Sheldrick, 1996)	*h* = −14→17
*T*_min_ = 0.422, *T*_max_ = 0.900	*k* = −9→10
13894 measured reflections	*l* = −18→16

### Refinement

**Table:**

Refinement on *F*^2^	Primary atom site location: structure-invariant direct methods
Least-squares matrix: full	Secondary atom site location: difference Fourier map
*R*[*F*^2^ > 2σ(*F*^2^)] = 0.036	Hydrogen site location: inferred from neighbouring sites
*wR*(*F*^2^) = 0.098	H atoms treated by a mixture of independent and constrained refinement
*S* = 1.04	*w* = 1/[σ^2^(*F*_o_^2^) + (0.0486*P*)^2^ + 0.4516*P*] where *P* = (*F*_o_^2^ + 2*F*_c_^2^)/3
3841 reflections	(Δ/σ)_max_ = 0.001
270 parameters	Δρ_max_ = 0.32 e Å^−^^3^
0 restraints	Δρ_min_ = −0.23 e Å^−^^3^

### Special details

**Table:**

Geometry. All e.s.d.'s (except the e.s.d. in the dihedral angle between two l.s. planes) are estimated using the full covariance matrix. The cell e.s.d.'s are taken into account individually in the estimation of e.s.d.'s in distances, angles and torsion angles; correlations between e.s.d.'s in cell parameters are only used when they are defined by crystal symmetry. An approximate (isotropic) treatment of cell e.s.d.'s is used for estimating e.s.d.'s involving l.s. planes.
Refinement. Refinement of *F*^2^ against ALL reflections. The weighted *R*-factor *wR* and goodness of fit *S* are based on *F*^2^, conventional *R*-factors *R* are based on *F*, with *F* set to zero for negative *F*^2^. The threshold expression of *F*^2^ > σ(*F*^2^) is used only for calculating *R*-factors(gt) *etc*. and is not relevant to the choice of reflections for refinement. *R*-factors based on *F*^2^ are statistically about twice as large as those based on *F*, and *R*- factors based on ALL data will be even larger.

### Fractional atomic coordinates and isotropic or equivalent isotropic displacement parameters (Å^2^)

**Table:**

	*x*	*y*	*z*	*U*_iso_*/*U*_eq_	
Br1	0.26620 (2)	0.56202 (4)	0.85086 (2)	0.06278 (16)	
S1	−0.08662 (5)	0.75087 (9)	0.66332 (5)	0.0568 (2)	
C9	−0.08406 (19)	0.4522 (3)	0.65402 (18)	0.0437 (7)	
H9A	−0.1163	0.3654	0.6481	0.052*	
C1	0.15871 (17)	0.5338 (3)	0.66735 (17)	0.0353 (6)	
H1A	0.1505	0.4901	0.7231	0.042*	
C5	0.00049 (18)	0.4509 (3)	0.62871 (16)	0.0347 (6)	
C6	0.06373 (18)	0.5807 (3)	0.63235 (17)	0.0361 (6)	
H6A	0.0694	0.6179	0.5746	0.043*	
C4	0.03938 (18)	0.3190 (3)	0.59218 (15)	0.0341 (6)	
N3	0.1653 (2)	0.1753 (3)	0.54913 (17)	0.0465 (7)	
C2	0.19932 (18)	0.4123 (3)	0.61042 (17)	0.0347 (6)	
C10	−0.02003 (18)	0.2040 (3)	0.56411 (17)	0.0400 (7)	
C3	0.12949 (18)	0.2955 (3)	0.58223 (16)	0.0351 (6)	
C11	0.2753 (2)	0.3418 (3)	0.66002 (19)	0.0447 (7)	
N4	−0.06658 (17)	0.1117 (3)	0.53989 (18)	0.0582 (7)	
C12	0.2385 (2)	0.4739 (3)	0.5331 (2)	0.0428 (7)	
C13	0.22316 (18)	0.6621 (3)	0.68104 (17)	0.0379 (6)	
C18	0.27173 (19)	0.6886 (3)	0.75675 (17)	0.0431 (7)	
C7	0.02957 (19)	0.7038 (3)	0.6874 (2)	0.0506 (8)	
H7A	0.0670	0.7893	0.6799	0.061*	
H7B	0.0361	0.6749	0.7465	0.061*	
N1	0.2686 (2)	0.5194 (3)	0.47443 (19)	0.0646 (8)	
N2	0.3323 (2)	0.2913 (3)	0.69975 (19)	0.0716 (8)	
C14	0.2323 (2)	0.7653 (3)	0.6164 (2)	0.0489 (8)	
H14A	0.2002	0.7527	0.5650	0.059*	
C8	−0.1330 (2)	0.5780 (3)	0.6909 (2)	0.0555 (8)	
H8A	−0.1309	0.5687	0.7522	0.067*	
H8B	−0.1961	0.5745	0.6713	0.067*	
C15	0.2877 (2)	0.8846 (3)	0.6273 (2)	0.0628 (9)	
H15A	0.2939	0.9498	0.5829	0.075*	
C17	0.3260 (2)	0.8109 (4)	0.7679 (2)	0.0633 (9)	
H17A	0.3570	0.8270	0.8196	0.076*	
C16	0.3338 (3)	0.9082 (4)	0.7027 (3)	0.0738 (11)	
H16A	0.3704	0.9901	0.7099	0.089*	
O1	0.65268 (16)	0.8365 (3)	0.50459 (17)	0.0758 (7)	
N5	0.53153 (19)	0.7245 (3)	0.55611 (19)	0.0675 (8)	
C19	0.6160 (2)	0.7654 (4)	0.5584 (3)	0.0671 (10)	
H19A	0.6517	0.7376	0.6056	0.081*	
C20	0.4938 (3)	0.6366 (6)	0.6229 (3)	0.1171 (17)	
H20A	0.5399	0.6179	0.6662	0.176*	
H20B	0.4721	0.5458	0.5995	0.176*	
H20C	0.4445	0.6882	0.6469	0.176*	
C21	0.4730 (3)	0.7652 (6)	0.4850 (3)	0.1229 (18)	
H21A	0.5064	0.8220	0.4460	0.184*	
H21B	0.4233	0.8218	0.5044	0.184*	
H21C	0.4501	0.6790	0.4571	0.184*	
H3A	0.131 (2)	0.119 (3)	0.5268 (18)	0.042 (9)*	
H3B	0.222 (3)	0.169 (4)	0.533 (2)	0.074 (12)*	

### Atomic displacement parameters (Å^2^)

**Table:**

	*U*^11^	*U*^22^	*U*^33^	*U*^12^	*U*^13^	*U*^23^
Br1	0.0733 (3)	0.0739 (3)	0.0399 (2)	−0.01304 (18)	−0.01097 (16)	0.00090 (16)
S1	0.0557 (5)	0.0529 (5)	0.0613 (5)	0.0176 (4)	−0.0045 (4)	−0.0084 (4)
C9	0.0404 (18)	0.0459 (17)	0.0447 (17)	−0.0014 (14)	0.0006 (14)	−0.0004 (13)
C1	0.0387 (15)	0.0341 (15)	0.0329 (15)	−0.0012 (12)	−0.0014 (12)	0.0010 (12)
C5	0.0372 (16)	0.0374 (16)	0.0289 (14)	0.0018 (12)	−0.0060 (12)	0.0021 (11)
C6	0.0403 (16)	0.0359 (16)	0.0317 (15)	0.0042 (12)	−0.0025 (12)	−0.0003 (12)
C4	0.0368 (16)	0.0320 (14)	0.0333 (14)	−0.0021 (12)	−0.0014 (12)	0.0026 (12)
N3	0.0385 (16)	0.0382 (16)	0.0624 (18)	−0.0015 (13)	−0.0031 (14)	−0.0120 (13)
C2	0.0353 (15)	0.0323 (15)	0.0359 (15)	0.0021 (12)	−0.0037 (12)	0.0026 (12)
C10	0.0356 (16)	0.0396 (17)	0.0450 (17)	0.0027 (14)	0.0039 (13)	0.0003 (13)
C3	0.0416 (17)	0.0286 (15)	0.0343 (15)	0.0002 (12)	−0.0055 (12)	0.0018 (12)
C11	0.0474 (18)	0.0373 (17)	0.0487 (18)	0.0025 (14)	−0.0066 (14)	−0.0090 (14)
N4	0.0469 (16)	0.0502 (16)	0.077 (2)	−0.0109 (14)	0.0022 (14)	−0.0160 (14)
C12	0.0422 (17)	0.0393 (17)	0.0467 (19)	−0.0041 (13)	−0.0005 (15)	−0.0043 (14)
C13	0.0391 (16)	0.0300 (15)	0.0444 (16)	0.0005 (12)	−0.0024 (13)	−0.0013 (13)
C18	0.0443 (17)	0.0378 (16)	0.0468 (17)	−0.0017 (13)	−0.0022 (14)	−0.0030 (13)
C7	0.0465 (18)	0.0455 (17)	0.059 (2)	0.0053 (14)	−0.0028 (15)	−0.0130 (15)
N1	0.075 (2)	0.0656 (19)	0.0541 (18)	−0.0172 (15)	0.0148 (16)	−0.0007 (15)
N2	0.070 (2)	0.0647 (19)	0.077 (2)	0.0193 (16)	−0.0305 (16)	−0.0106 (16)
C14	0.0499 (19)	0.0363 (17)	0.0597 (19)	−0.0021 (14)	−0.0066 (15)	0.0070 (15)
C8	0.0440 (18)	0.066 (2)	0.057 (2)	0.0067 (15)	0.0035 (15)	−0.0056 (16)
C15	0.063 (2)	0.0366 (18)	0.089 (3)	0.0007 (17)	0.005 (2)	0.0175 (18)
C17	0.060 (2)	0.053 (2)	0.076 (2)	−0.0118 (17)	−0.0147 (18)	−0.0157 (19)
C16	0.071 (3)	0.040 (2)	0.109 (3)	−0.0164 (17)	−0.012 (2)	−0.004 (2)
O1	0.0585 (16)	0.0812 (18)	0.0890 (19)	−0.0077 (13)	0.0197 (14)	−0.0008 (15)
N5	0.0437 (17)	0.084 (2)	0.075 (2)	0.0016 (15)	0.0046 (15)	0.0082 (17)
C19	0.054 (2)	0.074 (3)	0.073 (3)	0.0110 (19)	−0.0049 (19)	−0.013 (2)
C20	0.099 (4)	0.121 (4)	0.135 (4)	0.020 (3)	0.041 (3)	0.047 (3)
C21	0.074 (3)	0.190 (5)	0.103 (4)	−0.014 (3)	−0.022 (3)	0.025 (4)

### Geometric parameters (Å, °)

**Table:**

Br1—C18	1.890 (3)	C13—C18	1.389 (4)
S1—C8	1.788 (3)	C13—C14	1.402 (4)
S1—C7	1.795 (3)	C18—C17	1.385 (4)
C9—C5	1.328 (4)	C7—H7A	0.9700
C9—C8	1.494 (4)	C7—H7B	0.9700
C9—H9A	0.9300	C14—C15	1.372 (4)
C1—C13	1.523 (4)	C14—H14A	0.9300
C1—C6	1.546 (4)	C8—H8A	0.9700
C1—C2	1.568 (4)	C8—H8B	0.9700
C1—H1A	0.9800	C15—C16	1.363 (5)
C5—C4	1.468 (4)	C15—H15A	0.9300
C5—C6	1.512 (4)	C17—C16	1.371 (5)
C6—C7	1.525 (4)	C17—H17A	0.9300
C6—H6A	0.9800	C16—H16A	0.9300
C4—C3	1.364 (4)	O1—C19	1.216 (4)
C4—C10	1.431 (4)	N5—C19	1.302 (4)
N3—C3	1.339 (4)	N5—C21	1.436 (5)
N3—H3A	0.79 (3)	N5—C20	1.457 (5)
N3—H3B	0.89 (4)	C19—H19A	0.9300
C2—C12	1.485 (4)	C20—H20A	0.9600
C2—C11	1.488 (4)	C20—H20B	0.9600
C2—C3	1.539 (4)	C20—H20C	0.9600
C10—N4	1.145 (3)	C21—H21A	0.9600
C11—N2	1.128 (4)	C21—H21B	0.9600
C12—N1	1.126 (4)	C21—H21C	0.9600
			
C8—S1—C7	96.21 (14)	C6—C7—S1	113.2 (2)
C5—C9—C8	127.0 (3)	C6—C7—H7A	108.9
C5—C9—H9A	116.5	S1—C7—H7A	108.9
C8—C9—H9A	116.5	C6—C7—H7B	108.9
C13—C1—C6	112.8 (2)	S1—C7—H7B	108.9
C13—C1—C2	112.2 (2)	H7A—C7—H7B	107.7
C6—C1—C2	110.9 (2)	C15—C14—C13	121.5 (3)
C13—C1—H1A	106.8	C15—C14—H14A	119.2
C6—C1—H1A	106.8	C13—C14—H14A	119.2
C2—C1—H1A	106.8	C9—C8—S1	113.1 (2)
C9—C5—C4	121.0 (2)	C9—C8—H8A	109.0
C9—C5—C6	124.7 (2)	S1—C8—H8A	109.0
C4—C5—C6	114.3 (2)	C9—C8—H8B	109.0
C5—C6—C7	112.7 (2)	S1—C8—H8B	109.0
C5—C6—C1	110.2 (2)	H8A—C8—H8B	107.8
C7—C6—C1	108.6 (2)	C16—C15—C14	120.6 (3)
C5—C6—H6A	108.4	C16—C15—H15A	119.7
C7—C6—H6A	108.4	C14—C15—H15A	119.7
C1—C6—H6A	108.4	C16—C17—C18	119.9 (3)
C3—C4—C10	115.9 (2)	C16—C17—H17A	120.1
C3—C4—C5	125.2 (2)	C18—C17—H17A	120.1
C10—C4—C5	119.0 (2)	C15—C16—C17	119.8 (3)
C3—N3—H3A	117 (2)	C15—C16—H16A	120.1
C3—N3—H3B	124 (2)	C17—C16—H16A	120.1
H3A—N3—H3B	115 (3)	C19—N5—C21	119.4 (3)
C12—C2—C11	106.6 (2)	C19—N5—C20	122.4 (4)
C12—C2—C3	107.9 (2)	C21—N5—C20	118.2 (3)
C11—C2—C3	109.3 (2)	O1—C19—N5	126.2 (4)
C12—C2—C1	111.9 (2)	O1—C19—H19A	116.9
C11—C2—C1	107.8 (2)	N5—C19—H19A	116.9
C3—C2—C1	113.1 (2)	N5—C20—H20A	109.5
N4—C10—C4	178.4 (3)	N5—C20—H20B	109.5
N3—C3—C4	125.4 (3)	H20A—C20—H20B	109.5
N3—C3—C2	114.5 (2)	N5—C20—H20C	109.5
C4—C3—C2	120.1 (2)	H20A—C20—H20C	109.5
N2—C11—C2	177.7 (3)	H20B—C20—H20C	109.5
N1—C12—C2	179.4 (3)	N5—C21—H21A	109.5
C18—C13—C14	116.4 (2)	N5—C21—H21B	109.5
C18—C13—C1	123.6 (2)	H21A—C21—H21B	109.5
C14—C13—C1	119.9 (2)	N5—C21—H21C	109.5
C17—C18—C13	121.7 (3)	H21A—C21—H21C	109.5
C17—C18—Br1	116.2 (2)	H21B—C21—H21C	109.5
C13—C18—Br1	122.1 (2)		
			
C8—C9—C5—C4	−179.0 (3)	C11—C2—C3—C4	133.3 (3)
C8—C9—C5—C6	−0.4 (5)	C1—C2—C3—C4	13.2 (3)
C9—C5—C6—C7	13.9 (4)	C12—C2—C11—N2	121 (9)
C4—C5—C6—C7	−167.4 (2)	C3—C2—C11—N2	−123 (9)
C9—C5—C6—C1	135.4 (3)	C1—C2—C11—N2	1(9)
C4—C5—C6—C1	−46.0 (3)	C11—C2—C12—N1	40 (33)
C13—C1—C6—C5	−174.2 (2)	C3—C2—C12—N1	−77 (33)
C2—C1—C6—C5	58.9 (3)	C1—C2—C12—N1	158 (100)
C13—C1—C6—C7	−50.4 (3)	C6—C1—C13—C18	127.9 (3)
C2—C1—C6—C7	−177.3 (2)	C2—C1—C13—C18	−106.0 (3)
C9—C5—C4—C3	−164.3 (3)	C6—C1—C13—C14	−48.9 (3)
C6—C5—C4—C3	17.0 (4)	C2—C1—C13—C14	77.3 (3)
C9—C5—C4—C10	15.7 (4)	C14—C13—C18—C17	0.3 (4)
C6—C5—C4—C10	−163.0 (2)	C1—C13—C18—C17	−176.5 (3)
C13—C1—C2—C12	−47.4 (3)	C14—C13—C18—Br1	179.3 (2)
C6—C1—C2—C12	79.8 (3)	C1—C13—C18—Br1	2.4 (4)
C13—C1—C2—C11	69.6 (3)	C5—C6—C7—S1	−49.2 (3)
C6—C1—C2—C11	−163.2 (2)	C1—C6—C7—S1	−171.53 (19)
C13—C1—C2—C3	−169.4 (2)	C8—S1—C7—C6	62.6 (2)
C6—C1—C2—C3	−42.2 (3)	C18—C13—C14—C15	1.1 (4)
C3—C4—C10—N4	−48 (11)	C1—C13—C14—C15	178.1 (3)
C5—C4—C10—N4	131 (11)	C5—C9—C8—S1	22.8 (4)
C10—C4—C3—N3	−0.2 (4)	C7—S1—C8—C9	−47.3 (3)
C5—C4—C3—N3	179.8 (3)	C13—C14—C15—C16	−1.8 (5)
C10—C4—C3—C2	−179.9 (2)	C13—C18—C17—C16	−1.1 (5)
C5—C4—C3—C2	0.1 (4)	Br1—C18—C17—C16	179.9 (3)
C12—C2—C3—N3	69.1 (3)	C14—C15—C16—C17	1.0 (5)
C11—C2—C3—N3	−46.5 (3)	C18—C17—C16—C15	0.4 (5)
C1—C2—C3—N3	−166.6 (2)	C21—N5—C19—O1	−0.4 (6)
C12—C2—C3—C4	−111.2 (3)	C20—N5—C19—O1	178.7 (4)

### Hydrogen-bond geometry (Å, °)

**Table:**

*D*—H···*A*	*D*—H	H···*A*	*D*···*A*	*D*—H···*A*
N3—H3B···O1^i^	0.89 (4)	1.96 (4)	2.856 (4)	178 (3)
N3—H3A···N4^ii^	0.79 (3)	2.54 (3)	3.294 (4)	162 (3)
C15—H15A···O1^iii^	0.93	2.54	3.438 (4)	162

Symmetry codes: (i) −*x*+1, −*y*+1, −*z*+1; (ii) −*x*, −*y*, −*z*+1; (iii) −*x*+1, −*y*+2, −*z*+1.
